# Renal Denervation Promotes Atherosclerosis in Hypertensive Apolipoprotein E-Deficient Mice Infused with Angiotensin II

**DOI:** 10.3389/fphys.2017.00215

**Published:** 2017-04-13

**Authors:** Yutang Wang, Tam N. Dinh, Alexander Nield, Smriti M. Krishna, Kate Denton, Jonathan Golledge

**Affiliations:** ^1^School of Applied and Biomedical Science, Federation University AustraliaBallarat, VIC, Australia; ^2^The Vascular Biology Unit, Queensland Research Centre for Peripheral Vascular Disease, College of Medicine and Dentistry, James Cook UniversityTownsville, QLD, Australia; ^3^Cardiovascular and Renal Physiology, Department of Physiology, Monash UniversityClayton, VIC, Australia; ^4^Department of Vascular and Endovascular Surgery, The Townsville HospitalTownsville, QLD, Australia

**Keywords:** angiotensin II, aortic aneurysm, atherosclerosis, blood pressure, matrix metalloproteinase-2, renal denervation

## Abstract

**Objective:** To determine the effect of renal denervation (RDN) on the severity of atherosclerosis and aortic aneurysm in hypertensive mice.

**Methods:** Hypertension, atherosclerosis and aortic aneurysm were induced by subcutaneous infusion of angiotensin II (1 μg/kg/min) for 28 days in apolipoprotein E-deficient mice. RDN was conducted using combined surgical and local chemical denervation. The norepinephrine concentration in the kidney was measured by high-performance liquid chromatography. Blood pressure was measured by the tail-cuff method. Atherosclerosis was assessed by Sudan IV staining of the aortic arch. The aortic diameter was measured by the morphometric method. The mRNA expression of genes associated with atherosclerosis and aortic aneurysm were analyzed by quantitative PCR.

**Results:** RDN decreased the median norepinephrine content in the kidney by 93.4% (*n* = 5–7, *P* = 0.003) 5 days after the procedure, indicating that the RDN procedure was successful. RDN decreased systolic blood pressure in apolipoprotein E-deficient mice. Mice that had RDN had more severe aortic arch atherosclerosis (median percentage of Sudan IV positive area: 13.2% in control mice, *n* = 12, and 25.4% in mice having RDN, *n* = 12, *P* = 0.028). The severity of the atherosclerosis was negatively correlated with the renal norepinephrine content (spearman *r* = −0.6557, *P* = 0.005). RDN did not affect the size of aortic aneurysms formed or the incidence of aortic rupture in mice receiving angiotensin II. RDN significantly increased the aortic mRNA expression of matrix metalloproteinase-2 (MMP-2).

**Conclusion:** RDN promoted atherosclerosis in apolipoprotein E-deficient mice infused with angiotensin II associated with upregulation of MMP-2. The higher MMP-2 expression could be the results of the greater amount of atheroma in the RDN mice. The findings suggest further research is needed to assess potentially deleterious effects of RDN in patients.

## Introduction

Renal denervation (RDN) is used in clinical practice to lower blood pressure in treatment-resistant hypertension (Krum et al., 2009; Esler et al., 2010; Worthley et al., 2013; Bhatt et al., 2014; Papademetriou et al., 2014) by inhibiting the sympathetic outflow from the brain (Schlaich et al., 2009). RDN is generally regarded as a safe procedure (Krum et al., 2009; Esler et al., 2010; Bhatt et al., 2014). However, some studies have suggested that RDN may cause renal artery stenosis in 5–18% of patients (Kaltenbach et al., 2013; Worthley et al., 2013; Papademetriou et al., 2014; Versaci et al., 2014).

A number of experimental studies suggest that both chemical and surgical sympathetic denervation promote atherosclerosis (Murphy et al., 1957; Snyder and Campbell, 1958; Kacem et al., 1997, 2006; Kacem and Sercombe, 2008; Hachani et al., 2010). A recent study reported that RDN inhibited atherosclerosis formation in normotensive apolipoprotein E-deficient (ApoE^−/−^) mice fed a high-fat diet (Wang et al., 2015). The effects of RDN on atherosclerosis in mouse models that have hypertension has not however been investigated. This is important since RDN is performed in hypertensive patients. This study was designed to investigate whether RDN affects atherosclerosis severity in hypertensive ApoE^−/−^ mice infused with angiotensin II. As angiotensin II infusion also induces aortic aneurysm we also assessed the effect of RDN on aortic aneurysm severity.

## Methods

### Animals

Male ApoE^−/−^ mice (3 months old) were purchased from the Animal Resources Centre, Perth, Australia. All experiments were conducted in a temperature-controlled animal house (21 ± 1°C) under a 12:12-h light-dark cycle and mice were given standard chow and water *ad libitum*. All animal protocols conformed to the Guide for the Care and use of Laboratory Animals by the United States National Institutes of Health and the Australian Code of Practice for the Care and Use of Animals for Scientific Purpose (8th Edition, 2013). Institutional ethics approval was obtained from both James Cook University and Federation University Australia.

### Experimental protocol

A preliminary study was carried out to confirm the success of the RDN procedure. Five ApoE^−/−^ mice underwent sham surgery and seven mice underwent bilateral RDN. Five days later, the mice were euthanized and the right kidney was collected and norepinephrine content determined.

**Figure 1:**
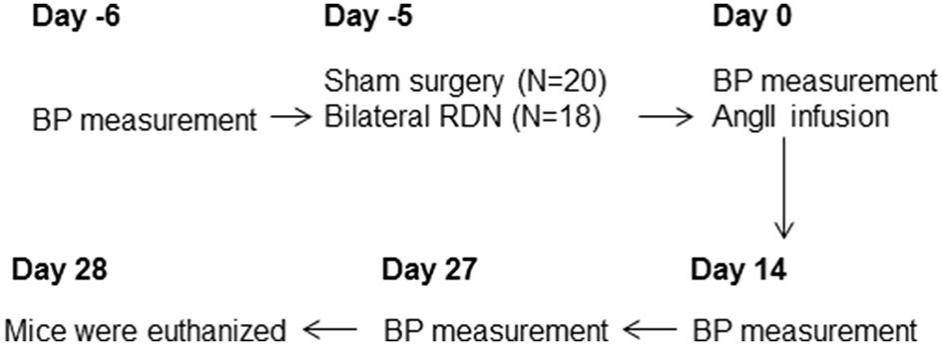
**A flowchart of the experiment**. The baseline blood pressure was measured followed by RDN or sham surgery on the next day. Five days after RDN or sham surgery, blood pressure was measured and all the mice were then subjected to angiotensin II infusion. Blood pressure was then measured at Day 14 and Day 27 during the angiotensin II infusion. The mice were euthanized at Day 28. Eight mice from the sham surgery group and six mice from the RDN group died of aortic rupture during the 28-day angiotensin II infusion. AngII, angiotensin II; BP, blood pressure; RDN, renal denervation.

For the main experiment, 18 ApoE^−/−^ mice underwent bilateral RDN and 20 ApoE^−/−^ mice underwent sham surgery 1 day after the baseline blood pressure was measured (Figure 1). Five days after RDN or sham surgery, blood pressure was measured and all the mice were then subjected to angiotensin II infusion at a dose of 1 μg/kg/min for the ensuing 28 days. Blood pressure was measured at Day 14 and Day 27 after the angiotensin II infusion commenced. The mice were euthanized at Day 28 (Figure 1) and the right kidney was collected for norepinephrine content measurement, and the aorta was isolated for morphometric analysis. The aortic arch was then used for Sudan IV staining and the thoracic aorta used for RNA extraction.

### Renal denervation

Bilateral RDN was carried out as previously described (O'Neill et al., 1991; Ye et al., 1997). In brief, after the renal arteries and veins were exposed, all visible nerves around the renal arteries were cut, and connective tissues passing next to and along the course of the renal arteries and veins were dissected and stripped off the adventitia under a dissecting microscope with a 4 × magnification. Then the renal arteries and veins were painted with a solution of 10% phenol in 95% ethanol (O'Neill et al., 1991; Ye et al., 1997). After being washed with saline (0.9% sodium chloride), the abdominal cavity was closed. For the mice in the sham surgery group, the renal arteries were exposed as with the RDN procedure, but the renal nerves were kept intact.

### Norepinephrine measurement

The right kidney was homogenized in 1 mM ethylenediaminetetraacetic acid (EDTA) and 4 mM sodium metabisulfite. The norepinephrine content in the homogenate was measured by phase isocratic high-performance liquid chromatography (HPLC) (Wang et al., 1999) coupled with an electrochemical detector, with an Atlantis C18 column (5 μm particle size, 4.6 × 150 mm) and a mobile phase of 50 mM Na_2_HPO_4_, 27 μM EDTA, 0.6 mM sodium octane sulfonic acid, and 3.5% acetonitrile (pH 4.0). This method had a good reproducibility with an inter-assay coefficient of variation of 4.7% (*n* = 10).

### Non-invasive tail-cuff blood pressure measurement

Blood pressure was measured using a computerized, non-invasive, tail-cuff system (Kent Scientific, USA) (Seto et al., 2013). Animals were habituated to the device before measuring blood pressure. Good reproducibility of this technique has been established previously (Seto et al., 2013).

### Induction of atherosclerosis and aortic aneurysm

Atherosclerosis and aortic aneurysm were induced by subcutaneous infusion of angiotensin II at a dose of 1 μg/kg/min for 28 days (Daugherty et al., 2000). Briefly, under general anesthesia using isoflurane, osmotic minipumps (Model 2004, ALZET, USA) were placed into the subcutaneous space along the dorsal midline to deliver 1 μg/kg/min of angiotensin (Sigma-Aldrich, Castle Hill, Australia) dissolved in distilled water over 28 days.

### Quantification of atherosclerotic lesion area

Atherosclerosis in the aortic arch was quantified by *en face* staining as described previously (Krishna et al., 2012). Briefly, the aortic arch was opened longitudinally and pinned down on a wax coated petri-dish. Tissue samples were transferred to a 70% ethanol solution and stained with 0.1% Sudan IV dissolved in equal parts of acetone and 70% ethanol for 10 min to identify areas of atherosclerosis. Sudan IV stained areas were quantified using Adobe Photoshop software (version CS5.1) and expressed as a percentage of the total aortic arch luminal surface area. We have previously established that these measurements can be repeated with good reproducibility (Golledge et al., 2010; Krishna et al., 2012).

### Measurement of the diameter of the aortic arch, thoracic, and suprarenal aorta

After the 28-day infusion with angiotensin II, mice were euthanized and aortas were harvested from their origin at the left ventricle to the iliac bifurcation, placed beside a ruler, and digitally photographed. The maximum diameters of the aortic arch, thoracic aorta and the suprarenal aorta were determined using the Adobe Photoshop CS5.1 software (Rush et al., 2009). We have previously established that these measurements can be repeated with good intraobserver reproducibility (Rush et al., 2009).

### Gene expression analysis

Six thoracic aortas were randomly selected from each group for RNA extraction. RNA was extracted using the TRI-reagent (Sigma-Aldrich, Castle Hill, Australia) in Eppendorf tubes according to the manufacturer's guidelines. The RNA yield of one sample from the sham surgery group was too low and not sufficient for the experiment, and thus this sample was not used for the subsequent cDNA synthesis and quantitative PCR. RNA was reverse transcribed to cDNA using the High Capacity Reverse Transcription Kit (Life Technologies). Gene expression was assessed by quantitative PCR using SYBR reagents. Primer sets are outlined in Table 1. The cycling conditions were as follows: a hold at 95°C for 2 min, followed by 40 cycles at 95°C for 15 s, 58°C for 20 s, and 72°C for 20 s. Relative gene expression was assessed using the 2^−ΔΔCt^ method (Livak and Schmittgen, 2001). Gene expression analysis was represented using relative gene expression compared with the control gene eukaryotic translation elongation factor 2 (EEF2) (Kouadjo et al., 2007).

**Table 1:** **Primer sets**.

**Gene**	**Primer Sets**	**Tm (°C)**	**Product length**
Adra2			
F	CAGCTCGCTGAACCCTGTTA	59.96	117
R	CACGATGCGTTTTCTGTCCC	60.04	
AT1A			
F	AGTTGGGAGGGACTGGATGA	59.88	149
R	GTTAAGTCCGGGAGAGCAGC	60.46	
AT1B			
F	GCAGGGAGTAACAGAGACCA	58.73	134
R	GTGAATTCAAAATGCACCCGT	57.97	
AT2			
F	TTTTAAGGAGTGCATGCGGGA	60.27	148
R	GGTAATGTTTCTGCTGGTGGC	59.8	
EEF2			
F	ACATGTCAGTCATCGCCCAT	59.46	166
R	GAGATGGCGGTGGATTTGATTG	59.97	
IL-6			
F	CGGCCTTCCCTACTTCACAA	59.68	149
R	GCCATTGCACAACTCTTTTCTCA	60.24	
iNOS			
F	CCTGCTTTGTGCGAAGTGTC	60.04	140
R	CCCTTTGTGCTGGGAGTCAT	59.96	
MCP-1			
F	CTTCTGGGCCTGCTGTTCA	59.93	127
R	CCAGCCTACTCATTGGGATCA	59.23	
MMP-2			
F	AACGGTCGGGAATACAGCAG	60.11	125
R	GTAAACAAGGCTTCATGGGGG	59.18	
MMP-9			
F	CAGCCGACTTTTGTGGTCTTC	59.74	87
R	ATAGCGGTACAAGTATGCCTCTG	59.99	
NF-κB			
F	GGCAGTGACGCGACGA	59.73	129
R	AAACAGATCGTCCATGGTCAGG	60.36	
TNF-α			
F	TAGCCCACGTCGTAGCAAAC	60.39	136
R	ACAAGGTACAACCCATCGGC	60.32	

*Adra2, α2 adrenergic receptors; AT1A, type 1A angiotensin receptor; AT1B, type 1B angiotensin receptor; AT2, type 2 angiotensin receptor; EEF2, eukaryotic translation elongation factor 2; F, forward; IL-6, Interleukin-6; iNOS, inducible nitric oxide synthase; MCP-1, monocyte chemoattractant protein-1; MMP, matrix metalloproteinase; NF-κB, Nuclear factor-kappa B; R, reverse; Tm, melting temperature; TNF-α, tumor necrosis factor-alpha*.

### Plasma cholesterol measurements

Blood was collected by cardiac puncture at the time of mice sacrifice. The concentration of total cholesterol, low-density lipoprotein/very low-density lipoprotein (LDL/VLDL) cholesterol and high-density lipoprotein (HDL) cholesterol in the plasma were quantified using a commercial available kit (Abcam, San Francisco, CA, USA; catalog number: ab65390) (Wang et al., 2014), according to the manufacturer's instructions.

### Statistical analyses

Continuous numbers were presented as a median and interquartile range (IQR). The difference between two groups was analyzed using Mann-Whitney *U*-test. Blood pressure was compared between mice that had RDN and controls during the experimental period using a linear mixed effect (LME) model using S Plus (software version 8.2). The difference in survival between the two groups was analyzed using log rank test. The correlation between atherosclerosis severity and renal norepinephrine content was assessed using the correlation analysis function of the GraphPad Prism 5 software (GraphPad Software, Inc., La Jolla, CA, USA). Differences were considered to be statistically significant at *P* < 0.05.

## Results

### Baseline parameters

The baseline body weight, systolic, diastolic and mean blood pressure, and the heart rate were similar in the sham surgery and the RDN groups (*P* > 0.05, Table 2).

**Table 2:** **The baseline parameters of the mice**.

**Group (sample size)**	**Body weight (g)**	**SBP (mm Hg)**	**DBP (mm Hg)**	**MAP (mm Hg)**	**Heart rate (beats/min)**
Sham surgery(*N* = 20)	29.6 (28.6–31.0)	101.8 (96.5–103.5)	81.0 (77.5–85.1)	87.7 (83.5–90.8)	502 (437–613)
RDN (*N* = 18)	30.4 (29.2–31.1)	101.5 (96.5–106.0)	79.8 (74.4–84.3)	86.0 (83.0–90.7)	422 (364–564)

*The baseline parameters were measured 1 day before the RDN or sham surgery procedures were conducted. Data were shown as a median and interquartile range (IQR). DBP, diastolic blood pressure; g, gram; MAP, mean arterial blood pressure; N, number; RDN, renal denervation; SBP, systolic blood pressure*.

### Success of the RDN procedure

**Figure 2:**
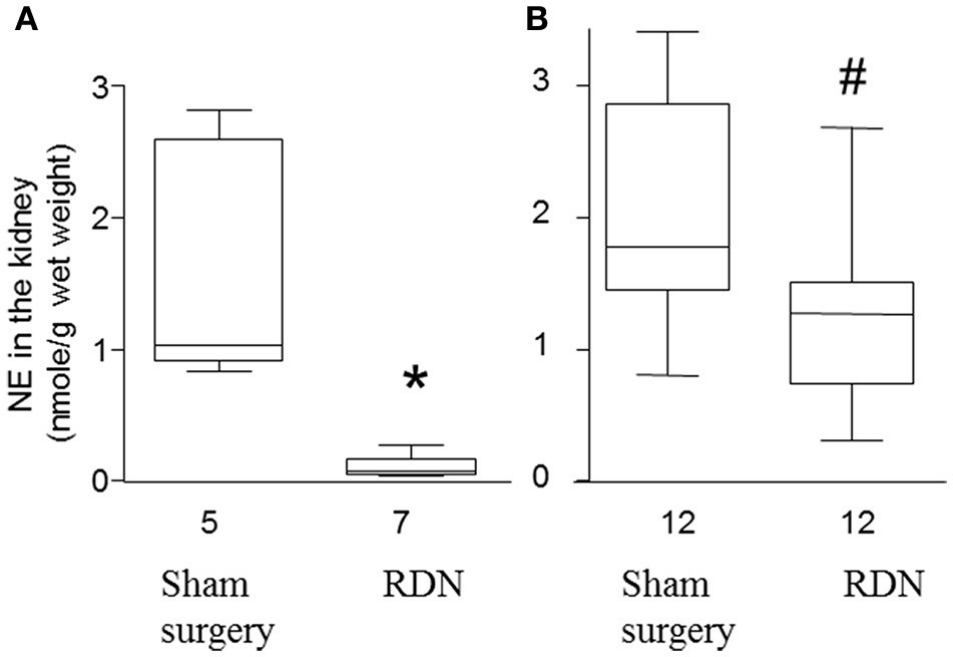
**Norepinephrine content in the kidney of apolipoprotein E-deficient mice. (A)** Mice were euthanized 5 days after sham surgery or RDN and the norepinephrine (NE) content in the kidney was determined. **(B)** Mice were euthanized after 28 days angiotensin II infusion and then the NE content in the kidney was determined. ^*^P = 0.003, ^#^*P* = 0.005, compared with sham surgery.

In a preliminary study, we performed RDN in seven mice and sham surgery in five mice. Five days later, mice were euthanized and norepinephrine content in the kidney was determined. RDN significantly decreased the median norepinephrine content in the kidney by 93.4% (*P* = 0.003, Figure 2A), which is similar to the previously reported ability of RDN to decrease norepinephrine content by 95% (Nakashima et al., 1996), suggesting that the RDN procedure was successful.

The norepinephrine in the kidney at the end of the main experiment (i.e., at the end of the 28-day angiotensin II infusion) was lower in the RDN group compared with sham surgery group (*P* = 0.005, Figure 2B). The median norepinephrine content in the RDN group was 71.7% of that in mice from the sham surgery group, suggesting a sustained reduction in renal innervation density.

### The effect of RDN on blood pressure

**Figure 3:**
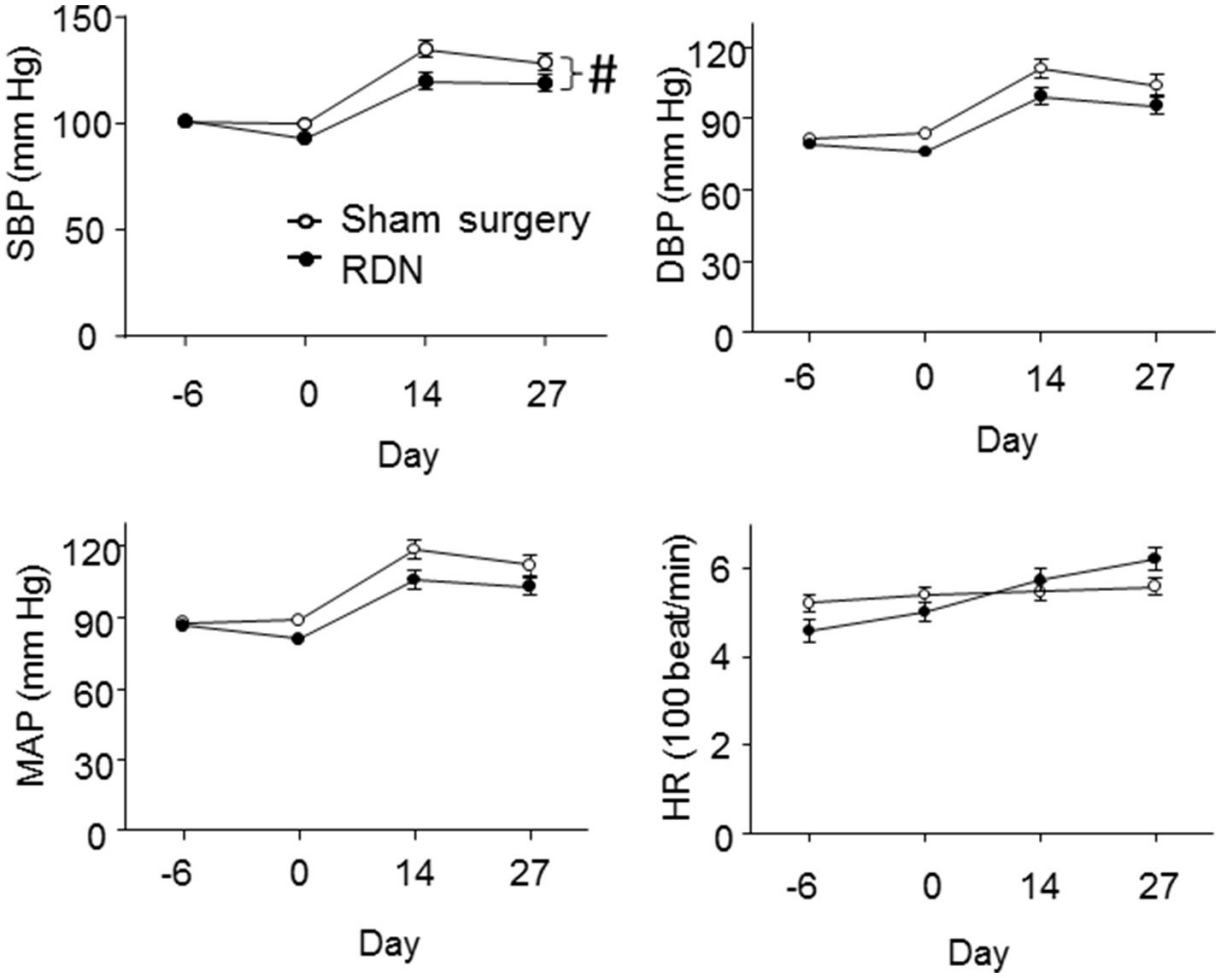
**Blood pressure and heart rate of the mice**. Blood pressure and the heart rate were measured by the tail-cuff method at baseline (Day -6), 5 days after RDN or sham surgery (Day 0), 14 days (Day 14) and 27 days (Day 27) after starting the angiotensin II infusion. SBP (#*P* = 0.017), but not DBP (*P* = 0.152) and MAP (*P* = 0.076), was significantly lower in mice having RDN compared to controls, as analyzed by LME models. DBP, diastolic pressure; HR, heart rate; MAP, mean arterial pressure; SBP, systolic blood pressure.

Baseline systolic, diastolic and mean blood pressure, and heart rate were not significantly different in mice prior to RDN and sham surgery (Figure 3). After RDN and sham surgery systolic blood pressure was significantly lower in mice that had RDN compared to controls (*P* = 0.017, Figure 3). Diastolic and mean blood pressure were not significantly different in mice having RDN and controls (Figure 3). RDN did not affect heart rate (*P* > 0.05, Figure 3).

### The effect of RDN on atherosclerosis

**Figure 4:**
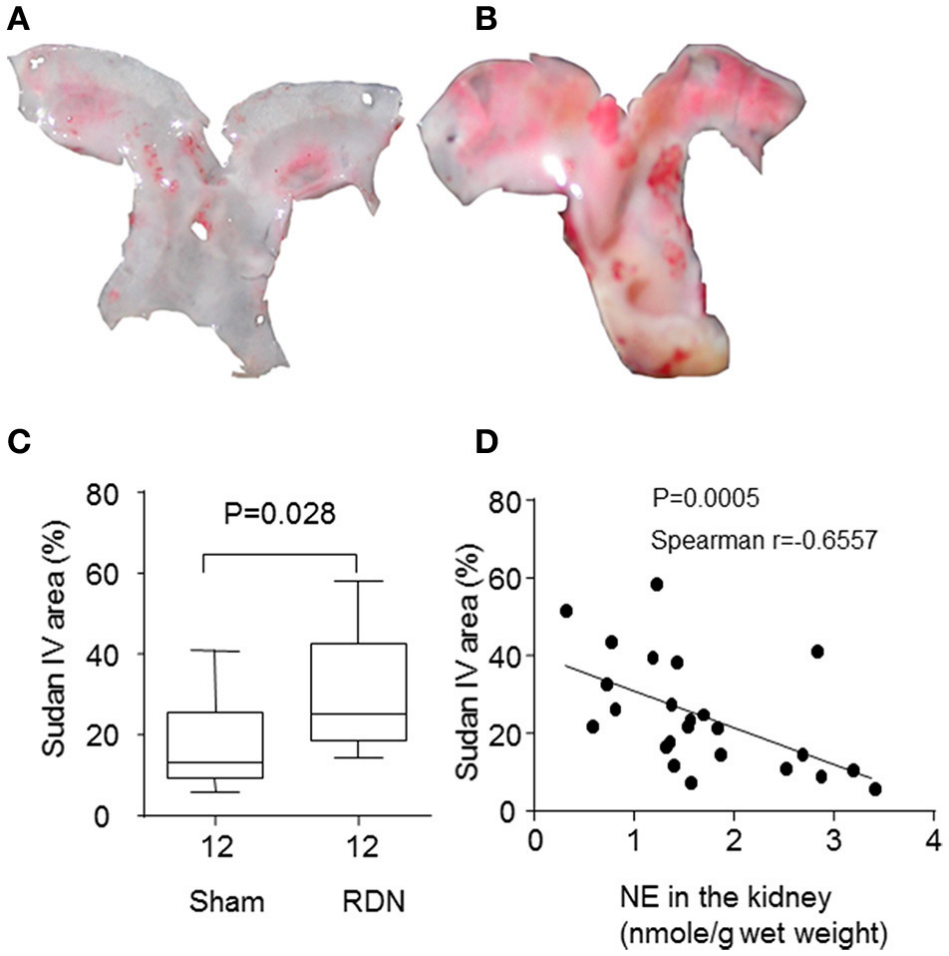
**RDN promoted more severe atherosclerosis in the apolipoprotein E-deficient mice infused with angiotensin II**. Mice were euthanized after infusion with angiotensin II for 28 days, and the aortas were collected. The surface of the aortic arch was stained *en face* with Sudan IV. **(A,B)** were representative Sudan IV images of the aortic arch from sham surgery and RDN groups, respectively. The percentage of Sudan IV-positive area was calculated and expressed as a percentage of the total aortic arch luminal surface area **(C)**. **(D)** The correlation between the norepinephrine (NE) content in the kidney and atherosclerosis in the aortic arch.

After 28 days of angiotensin II infusion, aortic arch Sudan IV staining area was significantly greater in the RDN group compared to the sham surgery group (*P* = 0.028, Figures 4A–C). In addition, the norepinephrine content in the kidney at the end of the experiment was negatively correlated with the Sudan IV staining area (Figure 4D).

### The effect of RDN on aortic aneurysm severity

**Figure 5:**
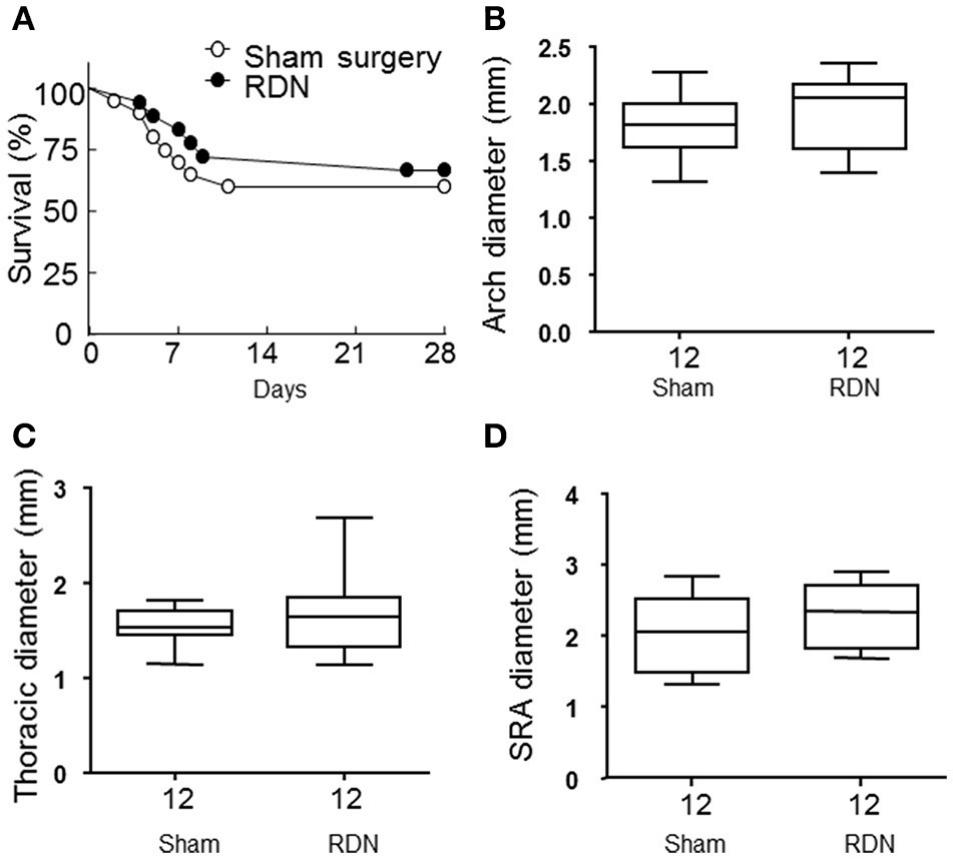
**RDN did not affect aortic rupture and the severity of the aortic aneurysm in the apolipoprotein E-deficient mice infused with angiotensin II. (A)** Mice in the sham surgery and RDN groups were subcutaneously infused with angiotensin II for 28 days. The death of mice was recorded during this period and the survival curves were then constructed. *P* = 0.299 between the two survival curves using log rank test. **(B–D)** Mice were euthanized after infusion with angiotensin II for 28 days. The aorta was then dissected for aortic aneurysm assessment. The maximal aortic diameter in the aortic arch **(B)**, thoracic **(C)**, and suprarenal (SRA, **D**) aorta were measured.

During the angiotensin II infusion, six mice died in the RDN group and eight died in the sham surgery group due to aortic rupture. There was no difference in the survival rate between the two groups (*P* > 0.05, Figure 5A). Angiotensin II infusion induced aortic aneurysm formation in the suprarenal and thoracic aorta (Krishna et al., 2015). The maximum diameter of the aortic arch, thoracic aorta and suprarenal aorta were not significantly different in mice receiving RDN and sham surgery (*P* > 0.05, Figures 5B–D).

### The effect of RDN on the aortic mRNA expression of some atherosclerosis associated genes

**Figure 6:**
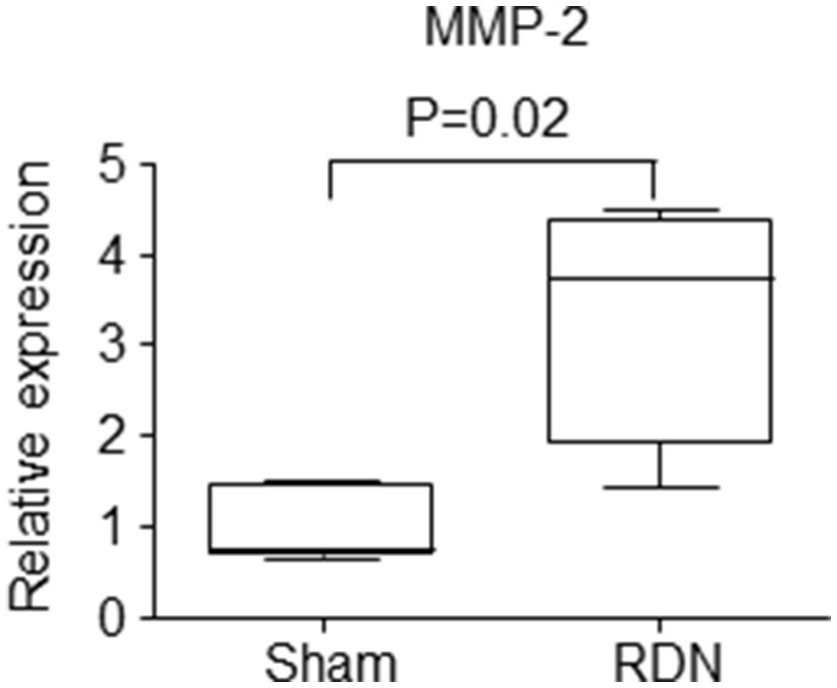
**The effect of RDN on the mRNA expression of MMP-2**. Mice were euthanized after infusion with angiotensin II for 28 days, and the thoracic aortic was used for RNA extraction. mRNA expression was analyzed using quantitative PCR. The relative gene expression was normalized using EEF2 as a reference gene. *N* = 5 for sham surgery, and *N* = 6 for RDN. MMP, matrix metalloproteinase.

mRNA expression of interleulin-6 (IL-6), inducible nitric oxide synthase (iNOS), monocyte chemoattractant protein-1 (MCP-1), nuclear factor-kappa B (NF-κB), tumor necrosis factor-alpha (TNF-α) and matrix metalloproteinase-9 (MMP-9) within the thoracic aorta were similar in mice receiving RDN and sham surgery (Table 3). Mice receiving RDN had higher thoracic aortic mRNA expression of MMP-2 than mice receiving sham surgery (Figure 6).

**Table 3:** **The effect of RDN on mRNA expression of pro-atherosclerosis markers, angiotensin receptors, and α2 adrenergic receptors**.

	**Sham**	**RDN**
AT1A	0.801 (0.735–1.364)	0.984 (0.574–1.295)
AT1B	0.834 (0.004–1.996)	0.039 (0.005–0.534)
AT2	0.803 (0.657–1.442)	1.669 (0.559–2.252)
Adra2	1.076 (0.784–1.178)	0.951 (0.546–1.293)
IL-6	1.011 (0.713–1.282)	0.501 (0.312–1.402)
iNOS	1.022 (0.741–1.0248)	1.311 (0.429–1.872)
MCP-1	0.633 (0.431–1.713)	0.433 (0.300–0.587)
NF-κB	0.951 (0.724–1.300)	0.890 (0.628–1.369)
MMP-9	0.969 (0.778–1.238)	1.472 (0.761–2.281)
TNF-α	0.863 (0.709–1.359)	0.992 (0.613–1.273)

*Mice were euthanized after infusion with angiotensin II for 28 days, and the thoracic aortic was used for RNA extraction. mRNA expression was analyzed using quantitative PCR. The relative gene expression was normalized using EEF2 as a reference gene. Data were expressed as median and interquartile range. N = 5 for sham surgery, and N = 6 for RDN. P > 0.05 for all the genes tested in the table. Adra2, alpha-2 adrenergic receptor; AT1A, type 1A angiotensin receptor; AT1B, type 1B angiotensin receptor; AT2, type 2 angiotensin receptor; IL, interleukin; iNOS, inducible nitric oxide synthase; MCP-1, monocyte chemoattractant protein-1; MMP, matrix metalloproteinase; NF-κB, nuclear factor-kappa B; RDN, renal denervation; TNF-α, tumor necrosis factor-alpha*.

### The effect of RDN on the aortic mRNA expression of angiotensin receptors and adrenoceptors

mRNA expression of angiotensin receptors (both type 1 and 2) and α2 adrenoceptors in the thoracic aorta were similar in mice that received RDN and sham surgery (Table 3).

### The effect of RDN on plasma cholesterol levels

**Figure 7:**
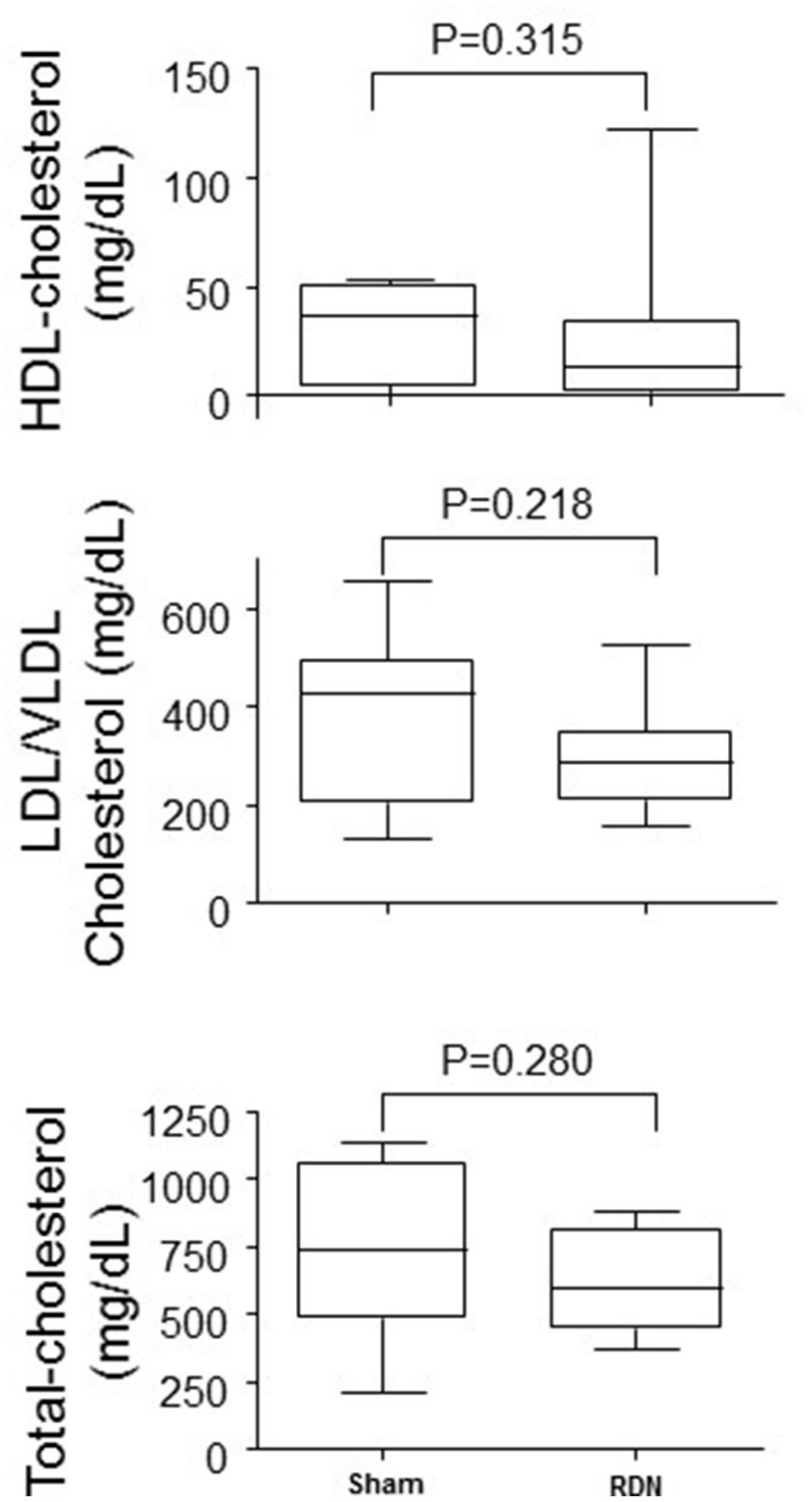
**Plasma lipid concentrations in mice undergoing RDN and controls**. Plasma lipid concentrations were measured in blood collected at the completion of the 28-day angiotensin II infusion. *N* = 10 for each group. HDL, high-density lipoprotein; LDL, low-density lipoprotein; VLDL, very low-density lipoprotein.

RDN did not affect plasma levels of LDL/VLDL cholesterol, HDL cholesterol, or total cholesterol (*P* > 0.05, Figure 7).

## Discussion

This study focussed on assessing the effect of RDN on atherosclerosis severity in a mouse model. RDN was successful performed as evidenced by substantially lower norepinephrine content of the kidney 5 and 33 days after the procedure and lower systolic blood pressure in mice having RDN compared to controls. The main finding of the study was that the severity of aortic arch atherosclerosis, as assessed by Sudan IV staining area, was greater in mice that received RDN than controls. The severity of atherosclerosis was correlated with the extent of the RDN, as assessed by renal norepinephrine levels. Our results suggest that RDN did not affect aortic aneurysm severity or rupture.

A number of previous experimental studies have reported that sympathetic denervation promoted atherosclerosis at sites remote to the denervation procedure (Murphy et al., 1957; Snyder and Campbell, 1958; Kacem et al., 1997, 2006; Kacem and Sercombe, 2008; Hachani et al., 2010). Bilateral surgical lumbar sympathectomy has been reported to increase atherosclerosis severity in the thoracic and abdominal aorta, and iliac and femoral arteries in rabbits fed a high cholesterol diet (Murphy et al., 1957; Snyder and Campbell, 1958). Similarly, sympathetic denervation induced by intravenous administration of 6-hydroxydopamine was reported to increase atherosclerosis within the basilar and femoral arteries of rabbits fed a high cholesterol diet (Kacem et al., 2006; Kacem and Sercombe, 2008). Sympathetic denervation by subcutaneous administration of guanethidine was reported to promote intima thickening within the abdominal aorta of rats fed a high cholesterol diet (Hachani et al., 2010).

The mechanisms underlying the atherosclerosis-promoting effect of sympathetic denervation are not well understood. It has been reported that this effect of sympathetic denervation may be due to stimulating migration of adventitial fibroblasts to the media and the associated loss and dedifferentiation of smooth muscle cells (Kacem and Sercombe, 2008; Hachani et al., 2010). It has been reported that following sympathetic denervation that the aortic expression of immature smooth muscle cell markers, such as vimentin, increased but that the expression of the mature smooth muscle cell markers (α-smooth muscle actin, h-caldesmon) decreased (Hachani et al., 2010). However, the importance of these changes in smooth muscle cell phenotype on the development of atherosclerosis have not been established.

Our results are in contrast to the results of a recent report which used another atherosclerotic model, i.e., ApoE^−/−^ mice fed a high-fat diet for 10 weeks (Wang et al., 2015). That study Wang et al. (2015) reported that RDN decreased atherosclerosis as assessed by oil-red-O staining within the aortic root and the aortic tree (including aortic arch, brachiocephalic artery, common carotid arteries and subclavian arteries). The discrepancy may be due to the disparate animal models used, i.e., high-fat diet vs. angiotensin II infusion in our study. The angiotensin II infusion model represents an advanced atherosclerosis model, as suggested by the large Sudan IV staining area reported in our study (Wang et al., 2015). In addition, the angiotensin II infusion model is a hypertensive model, whereas the high-fat diet model is a normotensive model. The latter model is different from the clinical setting as RDN is performed in treatment-resistant hypertensive patients.

MMP-2 expression within the thoracic aorta was greater in mice receiving RDN than controls. MMP-2 plays an important role in degrading extracellular matrix and has been implicated in the initiation, development and eventual rupture of atherosclerotic plaques (Li et al., 1996; Nagase and Woessner, 1999; Johnson et al., 2006; Kuzuya et al., 2006). It has been reported that MMP-2 protein and activity levels are increased in human aortic atherosclerotic lesions compared with normal regions of the aorta (Li et al., 1996). The severity of atherosclerosis in MMP-2 and ApoE double gene knockout mice has been reported to be less than that in ApoE single gene knockout mice (Kuzuya et al., 2006). The activity of MMP-2 and other MMPs can be inhibited by their endogenous tissue inhibitors (TIMPs) (Nagase and Woessner, 1999). It has been reported that over-expression of TIMP-2 by adenovirus technology significantly reduces atherosclerotic formation in the brachiocephalic artery of ApoE^−/−^ mice fed a high-fat diet (Johnson et al., 2006), and over-expression of TIMP-2 has also been reported to promote atherosclerotic plaque stability (Johnson et al., 2006). It is therefore possible that the upregulation of MMP-2 identified in mice receiving RDN may have promoted atherosclerosis within the aorta. It is also possible that the higher MMP-2 expression measured simply reflected the greater atherosclerosis in the mice receiving RDN although the fact other atherosclerosis-associated genes were not different supports a causative association. Further studies are needed to examine this theory. The expression of a range of other genes implicated in inflammation and matrix remodeling and plasma lipids were similar in mice receiving RDN and controls.

Other possible explanation for the greater atherosclerosis in mice receiving RDN include increased arterial pressure variability or functional changes within the media. These have not been investigated in the current study.

RDN has been reported to promote renal artery stenosis in humans. The Symplicity HTN studies (Krum et al., 2009; Esler et al., 2010; Bhatt et al., 2014) reported a renal artery stenosis rate of 0.3–2.2%, whereas other trials with smaller sample sizes have reported a higher rate of 2.8–18% (Kaltenbach et al., 2013; Worthley et al., 2013; Papademetriou et al., 2014; Persu et al., 2014; Versaci et al., 2014). The EnligHTN I trial (Worthley et al., 2013) reported that the progression of pre-existing renal artery stenosis was possibly related to the RDN procedure. Atherosclerosis is responsible for most primary renal artery stenoses but those developing after RDN may represent a local intimal hyperplasia response to the procedure rather than promotion of pre-existing atherosclerosis as identified in the mouse model studies here (Lao et al., 2011). Whether the renal artery stenoses reported in these trials are related to the promotion of atherosclerosis within the distant site of the aortic arch found in the current mouse study is not clear.

Renal denervation has been shown to decrease blood pressure in a large number of clinical (Krum et al., 2009; Esler et al., 2010) and preclinical (Nishihara et al., 2016) studies. However, the recent blinded, sham-controlled, randomized Symplicity HTN-3 trial (Bhatt et al., 2014) and a number of non-randomized trials (Brinkmann et al., 2012; Vase et al., 2012; Fadl Elmula et al., 2013; Hart et al., 2013; Ezzahti et al., 2014) suggested that RDN did not decrease blood pressure, and this may be due to an ineffective RDN procedure (Mahfoud et al., 2015). Currently there are no well-defined ways to immediately tell whether RDN has been technically successful in patients (Mahfoud et al., 2015). RDN was successful in our experiment as indicated by a substantial decrease in the renal norepinephrine content, and this was associated with a decrease in systolic blood pressure. RDN in our study did not affect heart rate, which is consistent with other reports (Bhatt et al., 2014; Vink et al., 2014).

### Limitations

This study has several limitations. First, the sample size of the study was small; second, this study employed only one animal model; third, blood pressure was measured by the tail-cuff method rather than the gold standard telemetry method; and fourth, the RDN procedure in mice is different from the clinical practice in which catheter-based methods are used. Finally the plasma concentrations of lipids varied substantially in different mice and the reasons for this are not clear.

### Potential clinical implication

Based on our results and other experimental evidence (Murphy et al., 1957; Snyder and Campbell, 1958; Kacem et al., 1997, 2006; Kacem and Sercombe, 2008; Hachani et al., 2010), there is concern that RDN might promote more severe distant atherosclerosis severity. Clinical studies are needed to examine this possibility in patients undergoing RDN.

## Author contributions

All authors listed, have made substantial, direct and intellectual contribution to the work, and approved it for publication.

### Conflict of interest statement

The authors declare that the research was conducted in the absence of any commercial or financial relationships that could be construed as a potential conflict of interest.
